# Supplementary material for: System analysis of cross-talk between nuclear receptors reveals an opposite regulation of the cell cycle by LXR and FXR in human HepaRG liver cells
Source: PLoS One. 2019 Aug 22;14(8):e0220894. doi: 10.1371/journal.pone.0220894 (PMC6705839; doi:10.1371/journal.pone.0220894)
Supplement: S1 Fig — (PDF) [file pone.0220894.s001.pdf]

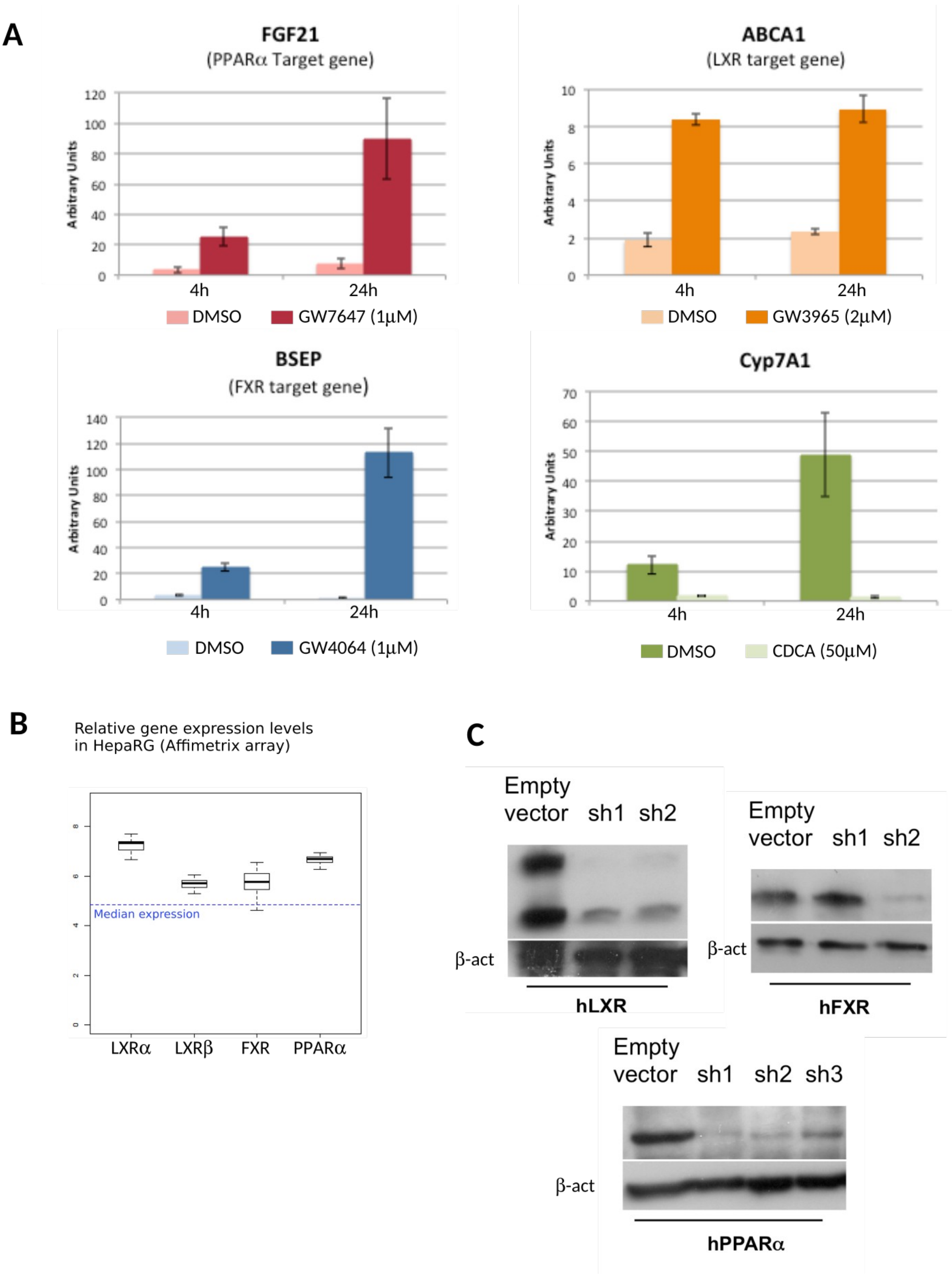


**S1 Fig. Expression and activity of LXR, FXR and PPAR in HepaRG cells.**
**(A)** Treatment of differentiated HepaRG cells by specific agonists for PPARα (top left), LXR (top right), and FXR (bottom left), and by the natural FXR agonist CDCA (bottom right), or by the vehicle (DMSO), as indicated. The response of a well-characterized target gene for each of the receptor, indicated at the top of each panel, has been assessed by qPCR. All cultures have been performed in three biological replicates. **(B)** Relative levels of LXRα, LXRβ, FXR, and PPARα expression at mRNA levels, as evaluated by microarrays. Each box-plot is generated from the values observed in the total of 36 microarrays performed in the study. **(C)** Western-blot performed with HepaRG cellular extract, following infection with a control adenovirus (Empty vector) or an adenovirus expressing an anti-sense RNA (sh) for LXR, FXR or PPARα, as indicated. The specific antibodies against LXR, FXR, and PPARα identify the band specific for each receptor. β-actin is used as loading control.
