# Supplementary material for: System analysis of cross-talk between nuclear receptors reveals an opposite regulation of the cell cycle by LXR and FXR in human HepaRG liver cells
Source: PLoS One. 2019 Aug 22;14(8):e0220894. doi: 10.1371/journal.pone.0220894 (PMC6705839; doi:10.1371/journal.pone.0220894)
Supplement: S2 Fig — (PDF) [file pone.0220894.s002.pdf]

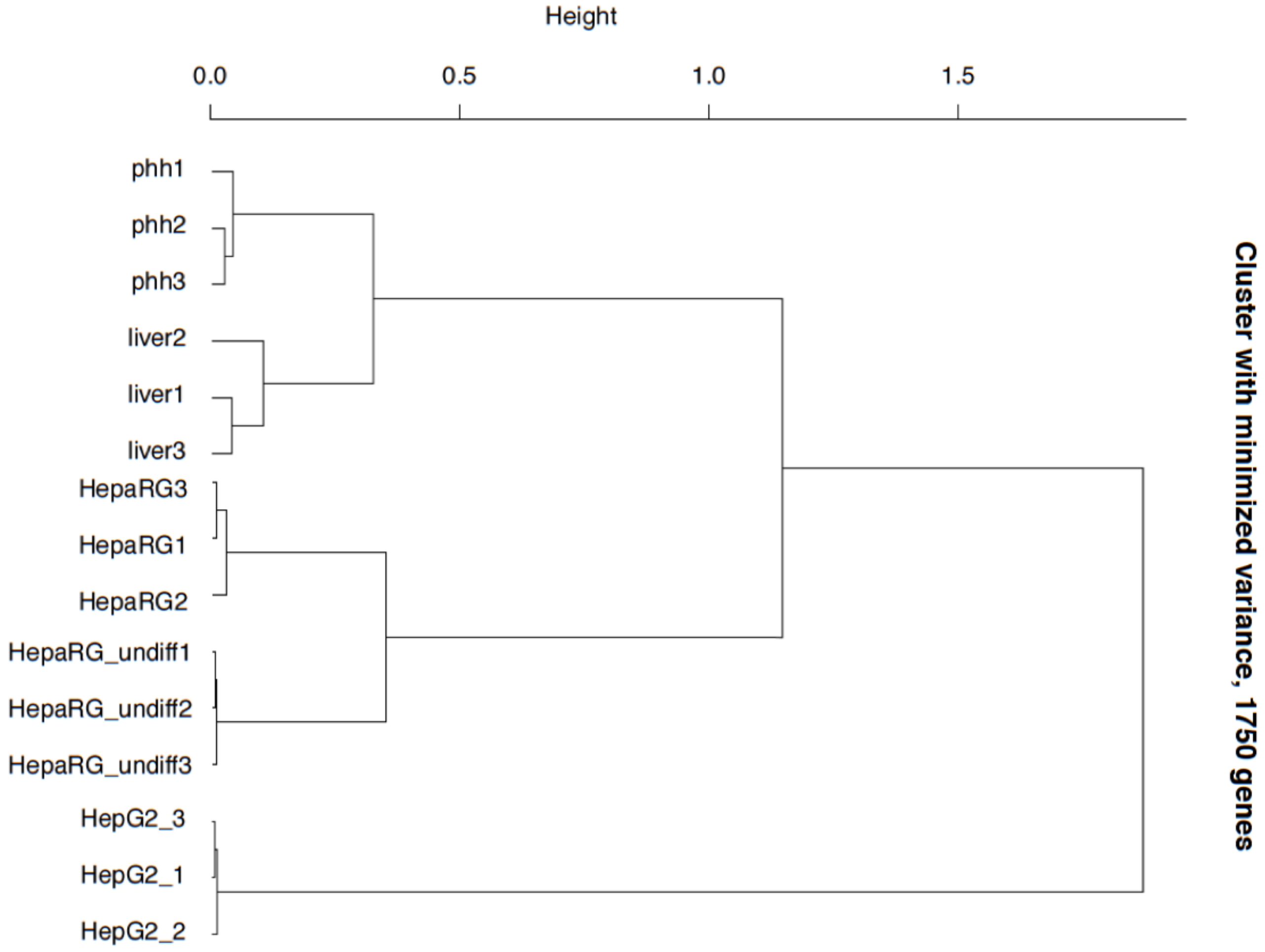


**S2 Fig. Hierarchical clustering of 15 microarray samples of liver cells and hepatic cell lines.**
This clustering is based on the publicly available Affymetrix data set from Hart et al.. Sample clustering was based on the expression profiles of the 1750 genes that were found significantly regulated by any of the four NR-treatments (FXR-L, CDCA, LXR-L, PPARα-L) in our own microarray study at either of the two time points (4h, 24h). As expected, primary hepatocytes (phh) are the closest to liver cells. Next closest are the HepaRG cells, while the HepG2 cells are clustering apart from all other cell types.
Significance testing for gene selection: Moderated F-tests with four NR-vs-control contrasts were performed separately per time point; p-values were adjusted by the Benjamini-Hochberg method; and genes with adjusted $p < 0.05$ in one or both of the two result lists were retained for the clustering analysis. Clustering method: Ward's minimum variance method, as implemented in the R function hclust prior to R version 3.0.3.
