# Supplementary material for: System analysis of cross-talk between nuclear receptors reveals an opposite regulation of the cell cycle by LXR and FXR in human HepaRG liver cells
Source: PLoS One. 2019 Aug 22;14(8):e0220894. doi: 10.1371/journal.pone.0220894 (PMC6705839; doi:10.1371/journal.pone.0220894)
Supplement: S3 Fig — (PDF) [file pone.0220894.s003.pdf]

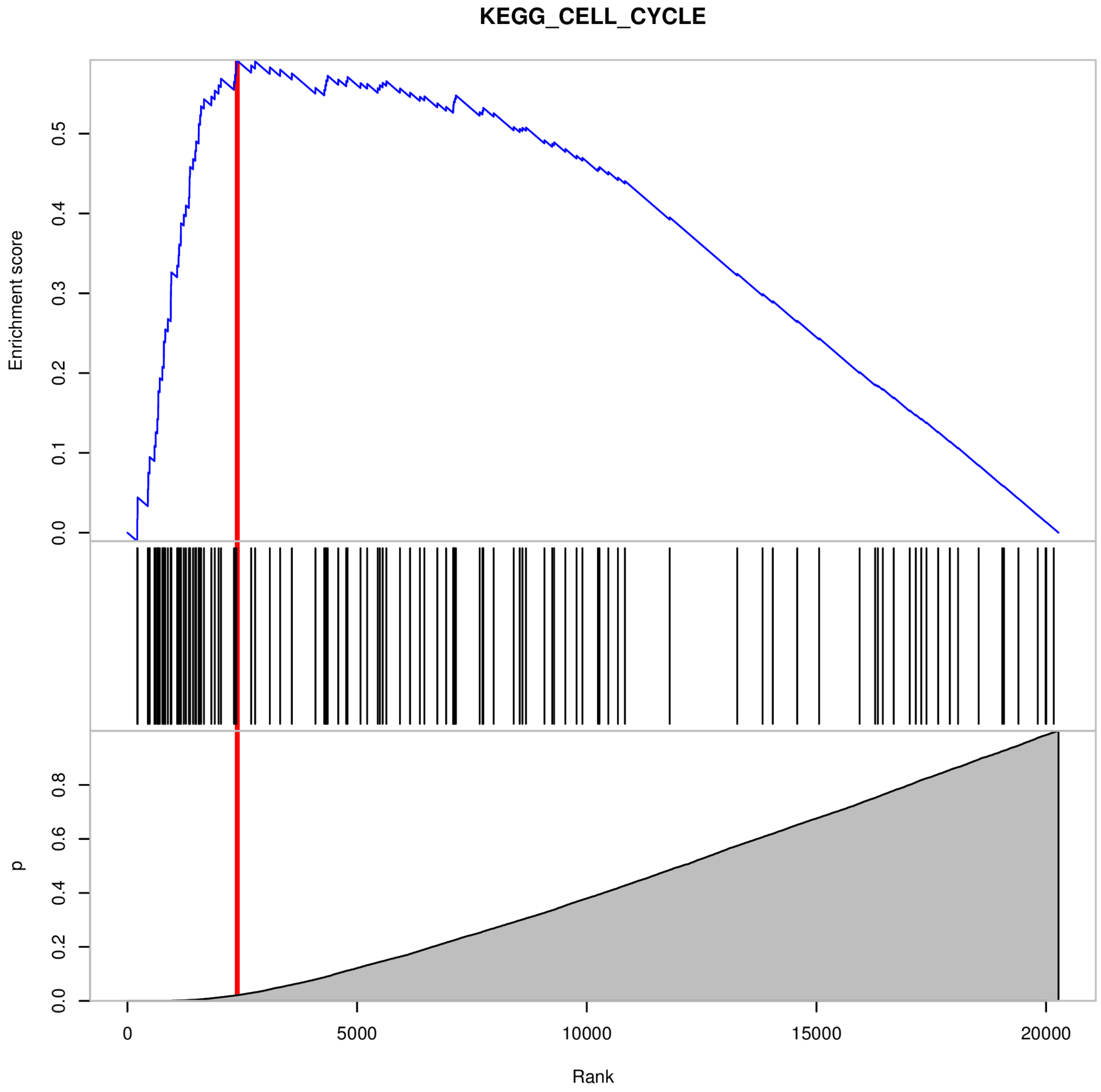

S3 Fig. Illustration of the GSEA method and selection of leading edge genes.
The horizontal axis represents genes, sorted according to their p-value obtained in a limma analysis (shown in the bottom panel). Genes associated to the pathway of interest (here the cell cycle) correspond to the vertical stripes in the center. On the top part, the blue curve represents the GSEA score, which increases when the current gene is part of the pathway and decreases otherwise (increasing and decreasing steps depend on the number of genes remaining to visit). The red vertical line marks the maximal score reached. The leading edge genes are the ones that have been visited before this line. The genes in over-represented (resp. under-represented) pathways have a higher density at the start (resp. end) of the list, which drives a high positive (resp. negative) score. The significance of these scores is assessed by permutations. See also Materials and Methods for more details.
