# Supplementary material for: System analysis of cross-talk between nuclear receptors reveals an opposite regulation of the cell cycle by LXR and FXR in human HepaRG liver cells
Source: PLoS One. 2019 Aug 22;14(8):e0220894. doi: 10.1371/journal.pone.0220894 (PMC6705839; doi:10.1371/journal.pone.0220894)
Supplement: S5 Fig — (PDF) [file pone.0220894.s005.pdf]

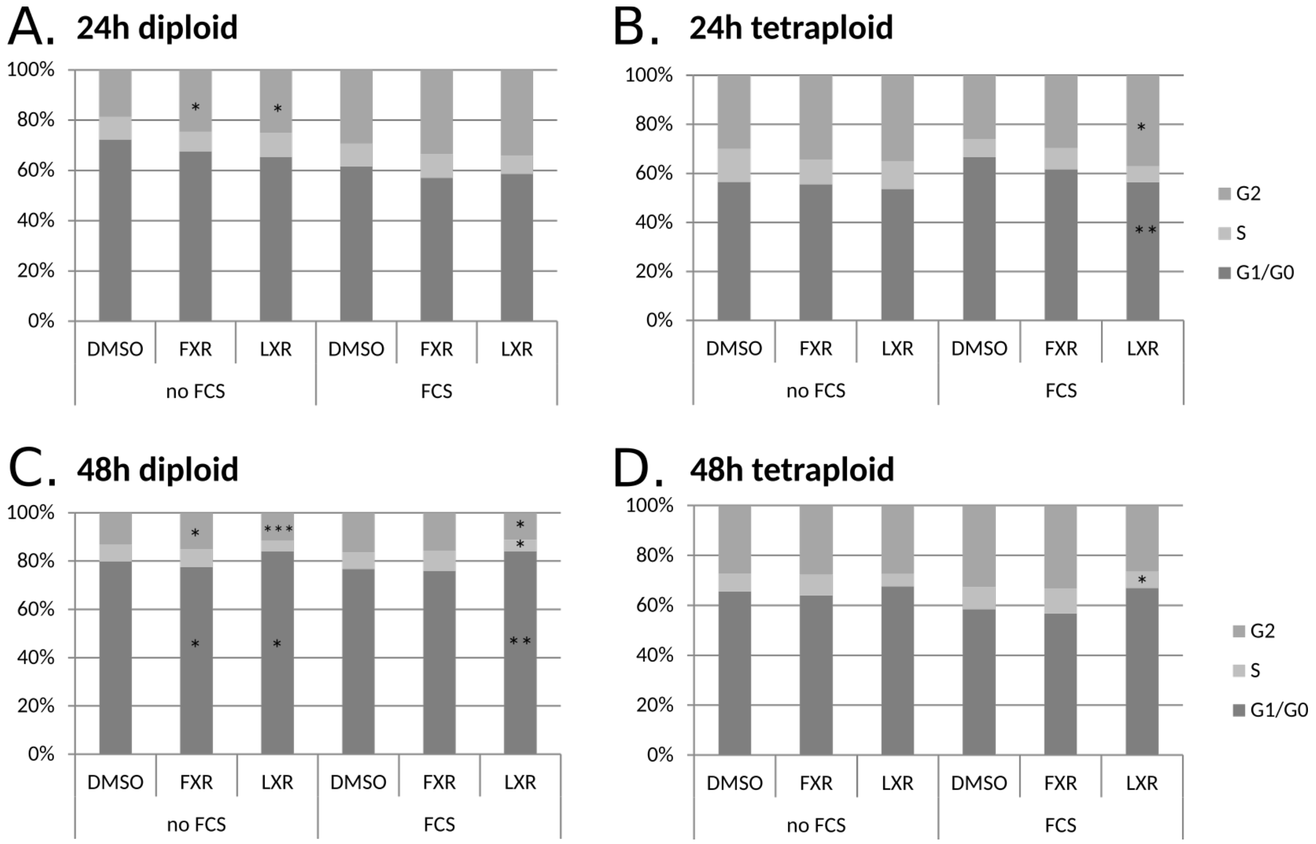

S5 Fig. FXR and LXR affect the cell progression and the ploidy of HepaRG cells.
The cell cycle distribution and the ploidy were analyzed by PI staining and FACS. **(A, B)** show the cell cycle distribution at 24h of treatment with DMSO, FXR-L 1 uM or LXR-L 2 uM. **(C, D)** show the cell cycle distribution at 48h with the different treatments. * : p-value < 0.05, ** < 0.01, *** < 0.001 versus DMSO (Student's t test). N=8.
