# Supplementary material for: System analysis of cross-talk between nuclear receptors reveals an opposite regulation of the cell cycle by LXR and FXR in human HepaRG liver cells
Source: PLoS One. 2019 Aug 22;14(8):e0220894. doi: 10.1371/journal.pone.0220894 (PMC6705839; doi:10.1371/journal.pone.0220894)
Supplement: S6 Fig — (PDF) [file pone.0220894.s006.pdf]

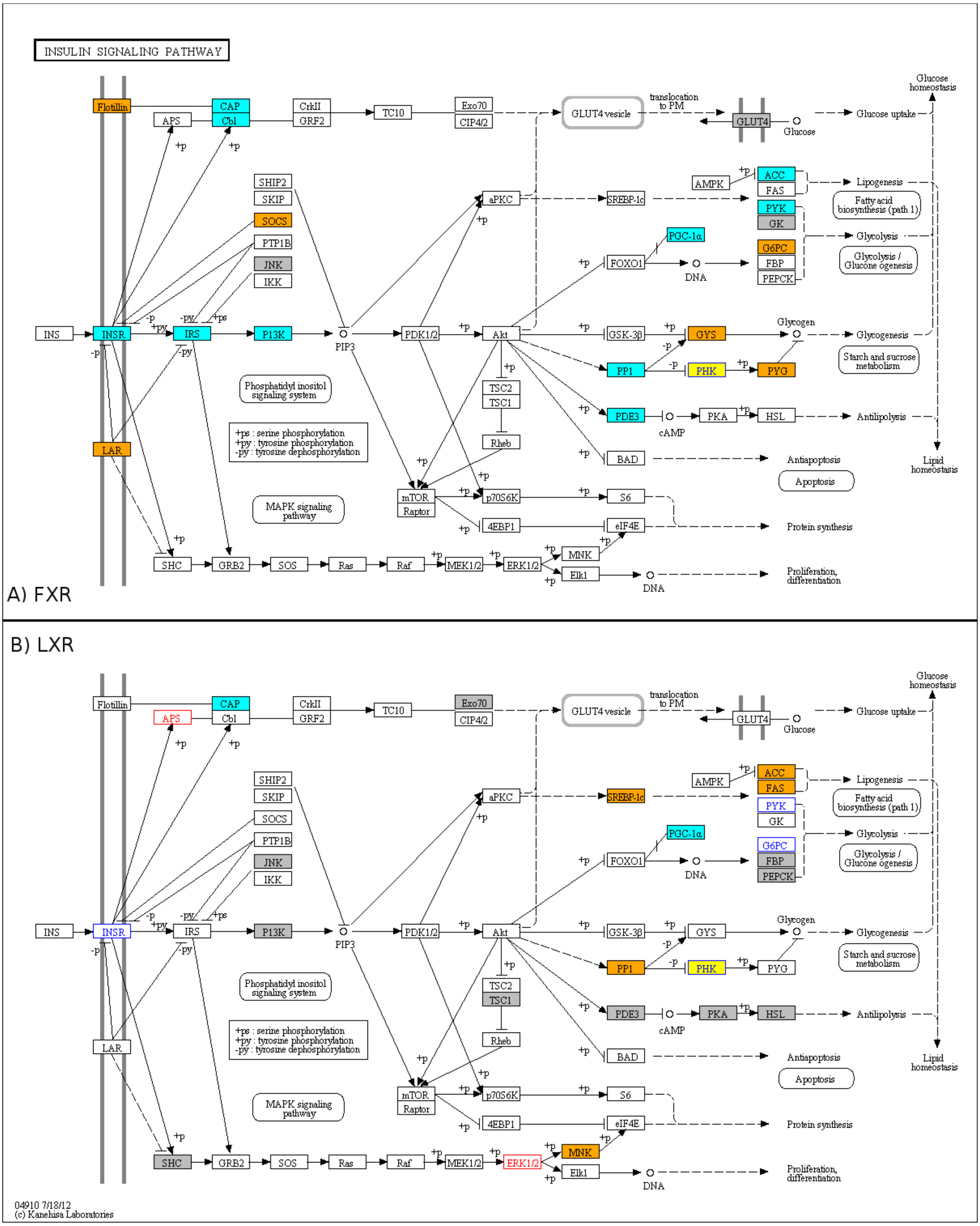

S6 Fig. Effect of the FXR-L and LXR-L treatments on the KEGG Insulin pathways.
(A) The genes in the leading edge for FXR-L treatment at 24h are highlighted, as in main Fig 3 (blue for down-regulations, orange for up-regulations). **(B)** Same image for the LXR-L treatment at 24h.
