# Supplementary material for: System analysis of cross-talk between nuclear receptors reveals an opposite regulation of the cell cycle by LXR and FXR in human HepaRG liver cells
Source: PLoS One. 2019 Aug 22;14(8):e0220894. doi: 10.1371/journal.pone.0220894 (PMC6705839; doi:10.1371/journal.pone.0220894)
Supplement: S1 Table — (PDF) [file pone.0220894.s007.pdf]

4h, FXR-L

Affymetrix Probe ID	**Gene Symbol**	**log2 Fold Change**	**Fold Change**	**t statistic**	**P-value**	**adj. P-value**
7966690	**TBX3**	1.48	2.80	13.39	2.14E-11	1.69E-08
8062823	**HNF4A**	-0.44	-1.35	-4.53	0.0002	0.01
8095628	**ALB**	-0.05	-1.04	-0.71	0.49	0.86
8012043	**ASGR1**	-0.09	-1.06	-0.41	0.68	0.93
8014591	**HNF1B**	-0.03	-1.02	-0.25	0.80	0.96

4h, CDCA

Affymetrix Probe ID	**Gene Symbol**	**log2 Fold Change**	**Fold Change**	**t statistic**	**P-value**	**adj. P-value**
7966690	**TBX3**	1.50	2.82	13.50	0.00	0.00
8062823	**HNF4A**	-0.14	-1.10	-1.48	0.15	0.87
8095628	**ALB**	-0.08	-1.05	-0.98	0.34	0.96
8014591	**HNF1B**	-0.06	-1.04	-0.55	0.59	0.99
8012043	**ASGR1**	-0.09	-1.07	-0.45	0.66	0.99

4h, LXR-L

Affymetrix Probe ID	**Gene Symbol**	**log2 Fold Change**	**Fold Change**	**t statistic**	**P-value**	**adj. P-value**
7966690	**TBX3**	0.21	1.15	1.88	0.08	1.00
8014591	**HNF1B**	0.08	1.06	0.79	0.44	1.00
8062823	**HNF4A**	0.06	1.05	0.67	0.51	1.00
8012043	**ASGR1**	-0.04	-1.02	-0.17	0.87	1.00
8095628	**ALB**	0.00	-1.00	-0.03	0.98	1.00

24h, FXR-L

Affymetrix Probe ID	**Gene Symbol**	**log2 Fold Change**	**Fold Change**	**t statistic**	**P-value**	**adj. P-value**
7966690	**TBX3**	1.23	2.34	15.09	4.47E-12	3.94E-09
8062823	**HNF4A**	-0.66	-1.58	-7.26	6.65E-07	6.99E-05
8012043	**ASGR1**	-1.05	-2.07	-5.78	0.00	0.00
8095628	**ALB**	-0.12	-1.09	-1.77	0.09	0.43
8014591	**HNF1B**	-0.10	-1.07	-0.59	0.56	0.87

24h, CDCA

Affymetrix Probe ID	**Gene Symbol**	**log2 Fold Change**	**Fold Change**	**t statistic**	**P-value**	**adj. P-value**
7966690	**TBX3**	0.84	1.79	10.31	3.00E-09	2.17E-06
8062823	**HNF4A**	-0.38	-1.30	-4.17	0.0005	0.03
8012043	**ASGR1**	-0.57	-1.48	-3.14	0.01	0.17
8095628	**ALB**	-0.05	-1.04	-0.73	0.47	0.92
8014591	**HNF1B**	-0.05	-1.04	-0.32	0.75	0.98

24h, LXR-L

Affymetrix Probe ID	**Gene Symbol**	**log2 Fold Change**	**Fold Change**	**t statistic**	**P-value**	**adj. P-value**
8062823	**HNF4A**	-0.39	-1.31	-4.27	0.00	0.10
7966690	**TBX3**	0.04	1.03	0.48	0.64	1.00
8012043	**ASGR1**	-0.07	-1.05	-0.40	0.69	1.00
8014591	**HNF1B**	-0.07	-1.05	-0.40	0.69	1.00
8095628	**ALB**	0.00	1.00	0.06	0.95	1.00

S1 Table. Expression profiles of hepatocyte differentiation markers.
Statistical test results for selected genes related to hepatocyte differentiation from the comparisons of ligand-treated cells and control cells (untreated and DMSO treated) at 4h and 24h. This table is composed of excerpts of the complete result tables from differential expression analysis, which are provided in the archive data_and_code.zip (data_and_code/genes). Results are from the differential gene expression analysis based described in Materials and Methods.
