# Supplementary material for: System analysis of cross-talk between nuclear receptors reveals an opposite regulation of the cell cycle by LXR and FXR in human HepaRG liver cells
Source: PLoS One. 2019 Aug 22;14(8):e0220894. doi: 10.1371/journal.pone.0220894 (PMC6705839; doi:10.1371/journal.pone.0220894)
Supplement: S2 Table — (PDF) [file pone.0220894.s008.pdf]

4h, FXR-L

Affymetrix Probe ID	**Gene Symbol**	**log2 Fold Change**	**Fold Change**	**t statistic**	**P-value**	**adj. P-value**
8131803	**IL6**	4.06	16.67	29.09	9.55E-18	1.94E-13
8054722	**IL1B**	0.98	1.97	8.6	4.01E-08	1.00E-05
7903786	**CSF1**	0.87	1.83	7.19	6.12E-07	9.40E-05
7980316	**TGFB3**	0.7	1.62	6.24	4.44E-06	0.00049209
7994280	**IL4R**	0.69	1.62	5.64	1.66E-05	0.00145666
8107887	**CSF2**	0.48	1.39	4.99	7.22E-05	0.00454671

S2 Table. IL6 and other proinflammatory cytokines upregulated by FXR-L at 4h.
Statistical test results for IL6 and a number of other proinflammatory cytokines from the comparison of FXR-L-treated cells and control cells (untreated and DMSO treated) at 4h. This table is an excerpt of the complete result table, which is provided in S1 File (genes/FXR_4h.txt). Results are from the differential gene expression analysis described in Materials and Methods.
