# Supplementary material for: System analysis of cross-talk between nuclear receptors reveals an opposite regulation of the cell cycle by LXR and FXR in human HepaRG liver cells
Source: PLoS One. 2019 Aug 22;14(8):e0220894. doi: 10.1371/journal.pone.0220894 (PMC6705839; doi:10.1371/journal.pone.0220894)
Supplement: S4 Fig — (PDF) [file pone.0220894.s004.pdf]

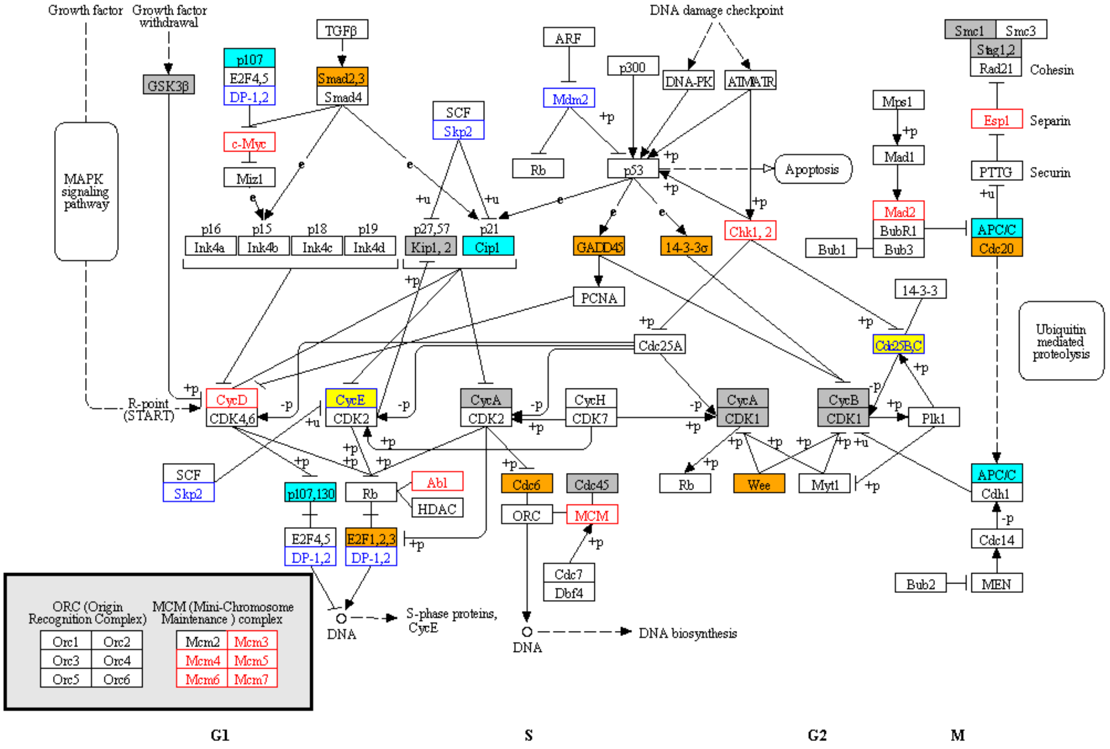

A. CDCA (4h)

B. FXR (4h)

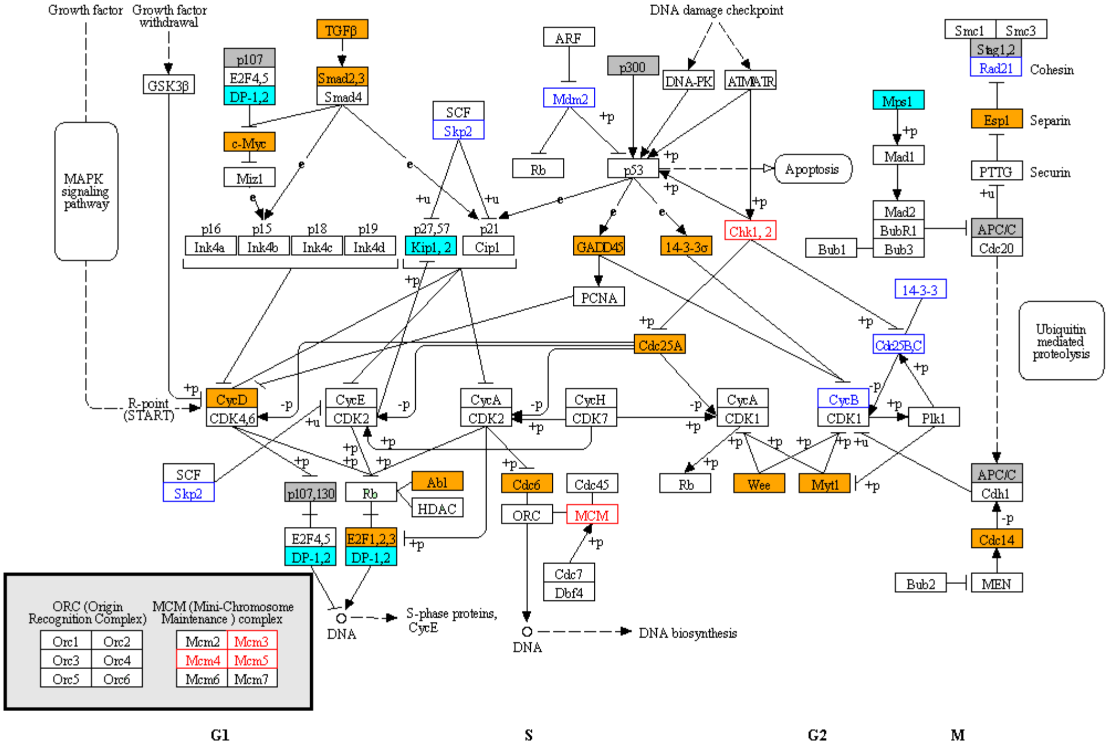

S4 Fig. Effect of the FXR-L and CDCA treatments on the KEGG cell cycle pathway at 4h.
(A) The genes in the leading edge for CDCA treatment at 4h are highlighted in blue for down-regulations and orange for up-regulations. **(B)** Same image for the FXR-L treatment at 4h.
